# Towards Latency Bypass and Scalability Maintain in Digital Substation Communication Domain with IEC 62439-3 Based Network Architecture

**DOI:** 10.3390/s22134916

**Published:** 2022-06-29

**Authors:** Lilia Tightiz, Joon Yoo

**Affiliations:** School of Computing, Gachon University, 1342 Seongnamdaero, Seongnam 13120, Korea; liliatightiz@gachon.ac.kr

**Keywords:** high-availability redundancy protocol (HSR), latency, parallel redundancy protocol (PRP), scalability, substation communication network

## Abstract

Parallel redundancy protocol (PRP) and high-availability redundancy protocol (HSR) are widely adopted protocols based on IEC 61850 standard to support zero recovery communication networks for time-critical and reliable interactions in power system substations. However, hiring these protocols comes with technical and economic constraints that impact the size of the substation network arrangement. Therefore, we will undertake a theoretical analysis of HSR, PRP, and their combinations to reach a maximum number of nodes in different substation communication architectures regarding IEC 61850 standard message time constraint requirements and IEC 62439-3 standard regulations. We will validate our findings through a simulation in the OPNET Modeler environment. In addition, we considered bandwidth efficiency by prohibiting the extra circulation of packets in the redundancy Box (RedBox) and QuadBox implementation as interfaces for HSR and PRP connection and HSR rings interconnection, respectively, which represent the main hindrance in utilizing the combination of these protocols.

## 1. Introduction

The introduction of serial communication to information exchange in the substation domain offered advantages such as increasing communication distance, cabling reduction, efficient bandwidth, and so forth. Several protocols using transmission control protocol (TCP)/(Internet protocol) IP over Ethernet have been hired for communications in substations, including Modbus, Profibus, distributed network protocol 3 (DNP3), and IEC 60870-5 [1,2]. Given that substations consist of versatile control and protection equipment manufactured by different companies, the requirement for a common language and interpretation of data exchange arose and was called interoperability. IEC 61850 protocol is an attempt started in 1996 by IEC Technical Committee 57 responding to interoperability for substation communication and matured over the following years to support in the yard and outside substations communication requirements. IEC 61850 divided substation communication into three levels, including process, bay, and station, and determine the latency, bandwidth, and other requirements for interaction within and between each level [3,4]. However, replacing hardwired-based interaction in the substation with Ethernet renders this data exchange procedure vulnerable to loss of connection, delay, traffic congestion, and packet queue. IEC 61850 predicted the solution for time and the reliable critical nature of interaction in the substation by offering parallel redundancy protocol (PRP) and high-availability redundancy protocol (HSR), which are defined by IEC 62439-3 standard [5]. Despite rapid spanning tree protocol (RSTP) as a hot-standby redundant protocol, HSR and PRP keep the redundant link active to ensure bumpless and reliable data exchange in substations. While PRP provides redundancy by duplicating each packet of information and conducting them to two independent local area networks (LAN) working in parallel, HSR implements the same concept in a ring network configuration using devices equipped with two-port links [6,7,8]. HSR and PRP have embedded in intelligent electronic devices (IED) located on the bay level and station level of substation communication which are responsible for monitoring, control, metering, and protection of substations by analyzing received information from sensors and actuators installed at the process level [9]. IEC 62439-3 standard extensively determines the specifications for each required piece of equipment for HSR and PRP implementation. PRP characterizes doubly attached node PRP (DANP) equipped with a pair of PRP-enabled switching ports sharing the same medium access control (MAC) address and upper layers. In the same line of thought but with a different operation, HSR specifies doubly attached node HSR (DANH) to establish ring network arrangement. Since HSR uses a modified Ethernet frame, legacy switches, servers, and workstations cannot connect directly to the HSR ring, and this protocol applies RedBox to solve this issue. QuadBox is another required node for the HSR ring that brings the ring of rings capability for HSR [10,11].

Each protocol has its advantages and disadvantages. The cost of HSR implementation is lower than PRP since PRP requires two independent LANs. In contrast, PRP offers n + 1 contingencies compared to HSR, which loses its ring arrangement when one of the ring nodes fails. In addition, PRP uses a standard Ethernet frame. As a result, PRP requires no special hardware while HSR has a specialized Ethernet frame incompatible with legacy network equipment. There is also a risk of packet flooding for the HSR ring with an excessive number of hops owing to the generation of duplicate packets in a single ring network infrastructure. This HSR performance mechanism, especially for SV, which produces periodic traffic, exacerbates the situation and reduces bandwidth efficiency. Using RedBox to connect HSR to PRP and QuadBox to provide cascading HSR rings presents another issue that threads the bandwidth by conducting extra traffic to other non-destination rings. Therefore, the deployed substation network architecture requires careful implementation of HSR, PRP, or their combination in substation communication network architecture based on IEC 62439-3 to achieve the tradeoff between each redundant method’s advantages and disadvantages.

Scholars have investigated substation redundancy arrangement in three areas, including drawbacks of HSR and PRP implementation improvement, protocol performance comparison, and substation network configuration optimization. Among them, there has been a surge of interest in the first two subjects.

Following the description of each HSR required equipment operation, R. Borgohain et al. [12] announced poor performances in latency, traffic, and bandwidth as dedicated HSR deficiencies. Quick removing and virtual ring were early attempts in HSR unicast surplus traffic reduction [13]. In the quick removing method, all nodes in an individual or connected HSR ring arrangement receive packets and drop any duplicates. Additionally, the virtual ring adopts the same approach as a virtual local area network (VLAN), which divides the ring into several rings and allows only relevant node traffic circulation in each virtual loop. I. Abdulsalam et al. [14] reduced the number of hops in HSR by providing a QuadBox ring connecting HSR rings. The authors, in this paper, deployed the port locking procedure to prevent conducting unicast packets in the non-destination HSR rings. Port locking includes two steps, namely the learning step and the working step. In the learning step, each QuadBox will learn the destination HSR ring to prohibit the extra circulation of the duplicate packet in the working stage. This approach still suffers from excessive traffic in the learning process. This idea was promoted in [15] by the HSR frame modification to provide a header for filtering traffic. In this method, QuadBox is equipped with two MAC address tables that assist the network in eliminating the circulation of the packet in non-destination HSR and QuadBox rings. Optimal dual paths is another technique utilized to reduce unicast HSR traffic by creating a separated dual connection between nodes before transmitting packets and optimizing this path based on link characteristics [16]. In further experiments [17], optimal dual virtual path solution boosted with dual virtual path and ring-based dual-path based on exchanging control messages between source and destination were employed to establish the dual path. To eliminate the complexity of hash tables, J. Araujo et al. [18] deployed segmented memory for detecting repeated packets. Sporadically MAC address detection following the add-drop multiplexer idea of SONET provided a remedy for the loop detection issue of HSR in [19]. The earlier approaches proposed in [20] were supplemented with a master ring arrangement to regulate rings of rings topology in order to offer scalability and failure management.

F. Steinhauser in [21] described PRP, HSR, and their benefits and drawbacks comprehensively. However, the findings did not support complementary modeling and simulations. S. Kumar et al. [22] examined revenue for the typical 132/22 kV substation protection system from PRP and HSR implementation. Through simulation on the OPNET Modeler, the authors proved that while both protocols fulfilled the time limitation of the protection system in failure conditions, PRP had a slight advantage in latency performance. The same results in HSR and PRP operation comparison acquired in [23] were achieved via hardware in loop execution for 275 kV substation architecture of a Scottish transmission power system.

Up to now, far too little attention has been paid to discovering constraints made by redundancy protocols on substation network arrangement size and optimizing the network performance with the combination of protocols. J. Larenzo et al. [24] audited the maximum number of nodes in an HSR ring respecting IEC 61850 message time delay specifications with and without MAC security overhead in communication frames. A full description of the ring, star, cascade, and mesh topology for IEC 61850-based substation communication is represented in [25] beyond the scope of providing redundancy with HSR and PRP arrangement.

The generation of fresh insights into readiness is required for planning substation communication networks concerning cost-effectiveness in deploying HSR and PRP. Therefore, in this study, we will undertake a theoretical analysis of HSR, PRP, and their combinations to achieve the maximum number of nodes in each architecture regarding IEC 61850 message time constraint requirements. The findings will be proved through a simulation in the OPNET Modeler environment. In addition, we considered bandwidth efficiency by prohibiting the extra circulation of packets in the implementation of RedBox and QuadBox as interfaces for HSR and PRP connection and connecting HSR rings, respectively, which represent the main hindrance in utilizing the combination of these protocols. Therefore, the main contributions of this paper are as follows:

- Investigation of a cost-effective substation network arrangement with a combination of HSR and PRP;
- Exploration of difficulties on deploying RedBox and QuadBox as principle equipment in HSR and PRP combination deployment;
- Determination of IEC 62439-3 based solution for our proposed arrangement’s obstacles;
- Theoretical and simulation analysis of nominated architectures concerning IEC 61850 communication constraints.

The outline of the paper is as follows. Section 2 clarifies the digital substation communication specifications mainly focus on latency. Section 3 specifies the HSR and PRP redundancy protocols according to IEC 62439-3. Section 4 proposes different configurations of substation’s communication with HSR and PRP and compares their performances. Ultimately, this paper finalizes in Section 5.

## 2. Digital Substations Latency Specifications

**Figure 1:**
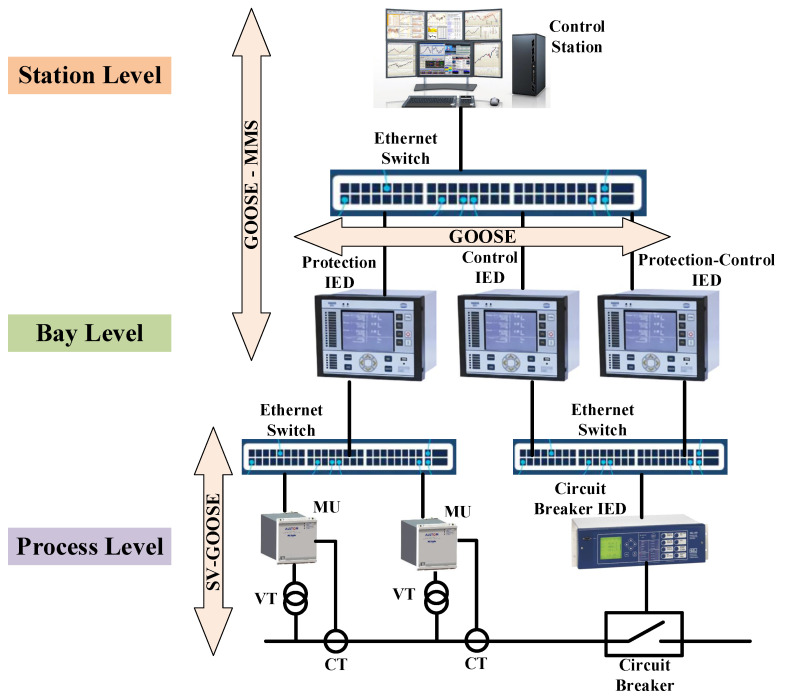
Communication levels and their relevant message types in a typical digital substation.

IEC 61850, as a commonly accepted protocol supporting interoperability in smart grid interactions, specifies three levels of communication inside substations, including process level, bay level, and station level. The station level is where the human–machine interface is located, and transfers required substation monitoring data to the work station and protection messages to the system operator. The bay level is the place where control and protection IEDs are installed and receive commands from the operator at the station level and measures data from current transformers (CT), voltage transformers (VT), merging units (MU), and circuit breakers located in the process level [26]. Manufacturing message specification (MMS), generic object-oriented substation event (GOOSE), and sampled value (SV) are messages that support interoperability between three communication levels of the substation, as shown in Figure 1. Low- or medium-level priority messages communicated with MMS type and GOOSE transfer critical protection messages. Therefore, MMS and GOOSE are two types of messages used at station and bay levels. SV is a message type with high priority that flows at the station level to transfer measurement information of metered devices to the bay level [27]. The other traffic between bay and station levels is the trip command from protection IEDs to circuit breakers and employs the GOOSE format.

Table 1 shows the specification of each message type. According to this table, the MMS transmission format is client/server and uses IP protocol, while GOOSE and SV are layer two traffic with multicast schema. It is noted that, in this paper, we considered the message size, sample frequency, and required throughput according to the IEC 61850 that is proposed in Table 1 [28].

**Figure 2:**
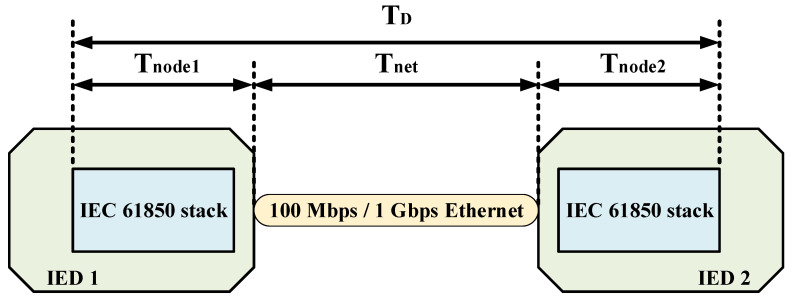
IEC 61850 ETE delay definition between two IEDs.

There are novel methods to calculate the end-to-end (ETE) delay for smart grid environment interactions [29,30]. Following the interoperability in digital substation communication, we consider the IEC 61850 the ETE delay of message transfer between IEDs calculation, represented in Figure 2 as follows [1].

(1) $$T_{D}=T_{node1}+T_{net}+T_{node2},$$

where $T_{D}$ is ETE delay time, and $T_{node}$ is data processing time in each IED calculated according to (2).

(2) $$T_{node}=T_{proc}+T_{sw}+T_{q},\;\;T_{q}\;\neq \;0\;\;if\;\;node\;\;is\;\;source,$$

where $T_{proc}$ is the time of processing frames by the node, $T_{sw}$ is the time it takes for the node to decide whether to accept the frame for forwarding or discard it, and $T_{q}$ is the time that the frame waits to forward. It is noted that $T_{q}$ occurs only on the source. $T_{net}$ is the network communication transfer time estimated by (3).

(3) $$T_{net}=T_{prop}+T_{Tran},$$

(4) $$T_{prop}=\frac{d}{v},$$

where *d* is communication link length and *v* is propagation speed and equal to 2 × $10^{8}$ (m/s) here.

(5) $$T_{Tran}=\left(P+N_{d}\right)\frac{L}{R},$$

where *P* is the preamble size, $N_{d}$ is the number of forwarding decision bytes, *L* is packet length, and *R* is link bandwidth (bps).

## 3. Redundancy Protocols Implementation Requirements Based on IEC 62439-3

### 3.1. PRP

**Figure 3:**
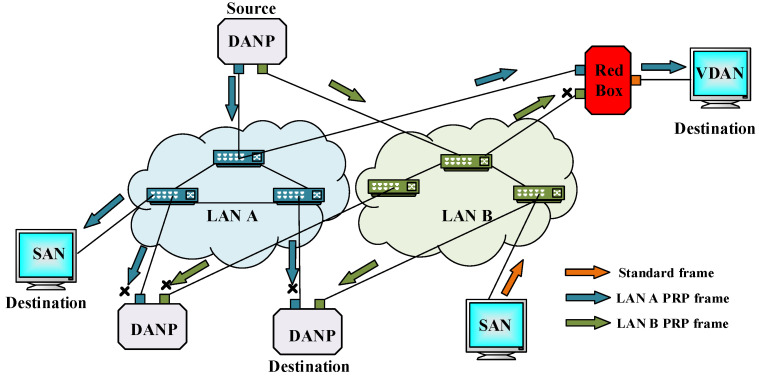
PRP performance.

To avoid latency in transferring critical data in the substations, PRP arranges a redundant network by implementing two parallel separated LANs with similar MAC addresses and the possibility of different topology and performances. Figure 3 shows the PRP network arrangements. This network should support several nodes with unique characteristics, including DANP, SAN, virtual doubly attached node (VDAN), and RedBox. The main component of each PRP node is DANP which enables nodes to connect to both parallel LAN networks. Every source duplicates the frames and propagates them over two parallel LANs in a PRP network. The DANPs on the destination side receive the first copy of the frame and discard the duplicate. When any one of the redundancy network components fail, this performance results in zero recoveries.

**Figure 4:**
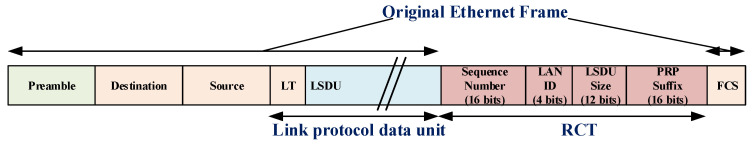
PRP frame.

The DANPs have two receiver and transmitter ports connected to the same upper layers by a link redundancy entity (LRE). The main tasks of LRE are the generation and removal of duplicate frames achieved by attaching or detaching the redundancy check trailer (RCT), respectively. The RCT, as shown in Figure 4, includes frame information, such as sequence number, LAN identifier, link service data unit (LSDU) frame size, and PRP suffix. The RedBox is auxiliary equipment used in both PRP and HSR networks. This device enables SAN to connect to the PRP or HSR. RedBox enhances the SAN to perform similar to a DAN and provides a VDAN. RedBox has a propriety IP address and establishes a DANP to play the role of LRE. RedBox, similar to DANP, has two pairs of sender and receiver ports and one or more ports to connect ordinary SAN devices. Due to RCT’s inclusion in PRP frames as a trailer, SANs can connect directly to the PRP network and communicate. Another advantage of the PRP is its scalability since it utilizes LANs that can support numerous nodes. PRP arrangement supports both station bus and process bus communication.

### 3.2. HSR

**Figure 5:**
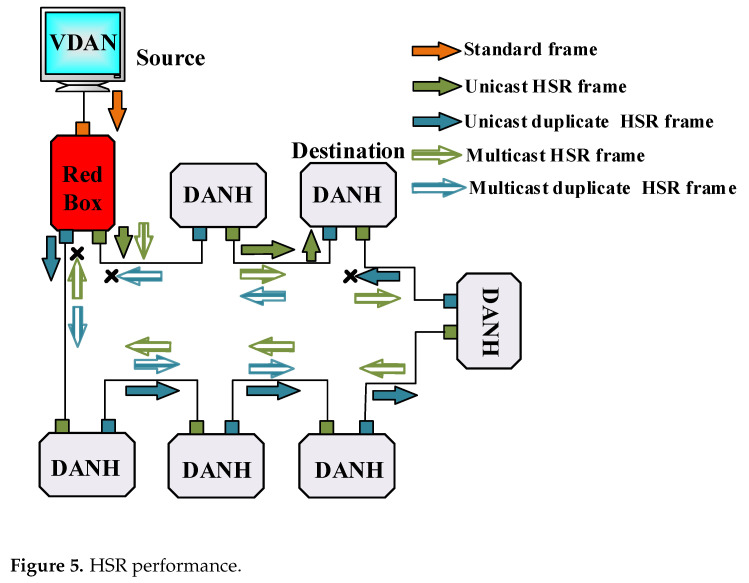
HSR performance.

HSR is another approach to providing a reliable Ethernet network, which contrary to PRP, does not require Ethernet switches to establish a ring structure and consequently offers cost-effective redundancy. As a result of this feature, the only means to link SAN to the HSR ring will be through RedBox, which has the same performance as indicated in Section 3.1. Therefore, HSR network is not scalable as PRP. RedBox is also used to connect PRP to the HSR network, and up to six PRP networks are permissible for connecting to the HSR. Nodes in HSR, called DANH, duplicate each frame in the source node, similar to DANP. However, in the HSR ring, since there is no separated parallel network such as PRP, it should pass a frame that is not its destination to provide a ring path. Figure 5 reveals that each DANH when receiving duplicate unicast messages frame, drops the copy one, and in multicast, it discards the repetitive frame in the source node.

**Figure 6:**
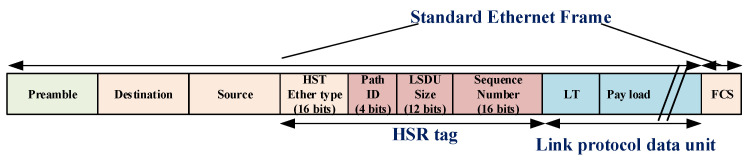
HSR frame.

The HSR tag, as shown in Figure 6, contains the HSR Ether type, path identifier, LSDU size, and sequence number. Path identifier with its three first bits specifies the PRP network destination to avoid traffic flooding to other PRP networks when the network architecture uses HSR with several PRP networks. The last bit of the network identifier shows the PRP network LAN ID, which can be A or B.

**Figure 7:**
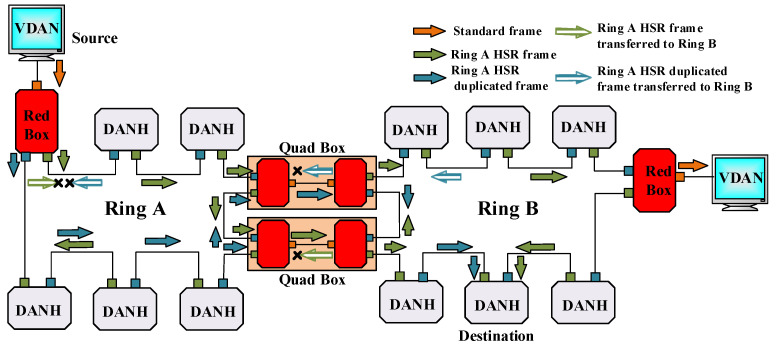
HSR ring extension with QuadBox.

The number of HSR rings extends by connecting HSR rings with QuadBoxes. Two RedBoxes that connect with a common interlink provide the QuadBox. The device forwards frames without modifying them from one HSR ring to another and can filter multicasts and VLAN traffics. The standard recommends using two QuadBoxes for HSR ring connection to prevent the risk of a single point of failure, as shown in Figure 7. However, each QuadBox, when receiving a frame from its interlink or mated QuadBox, sends the earlier arrived one and removes the repeating form of the frame. Deploying this configuration prevents four frame copies from being generated in each ring.

## 4. High-Availability Substation Network Configurations Comparison

This section examines different approaches for providing redundancy in substations’ communication networks. We initially implement DANH, DANP, RedBox, and QuadBox based on IEC 62439-3 on the OPNET Modeler and then determine the optimum number of nodes for the HSR, PRP, and their combinations considering scalability, latency, and financial aspects.

### 4.1. IEC 62439-3 Elements Simulation

**Figure 8:**
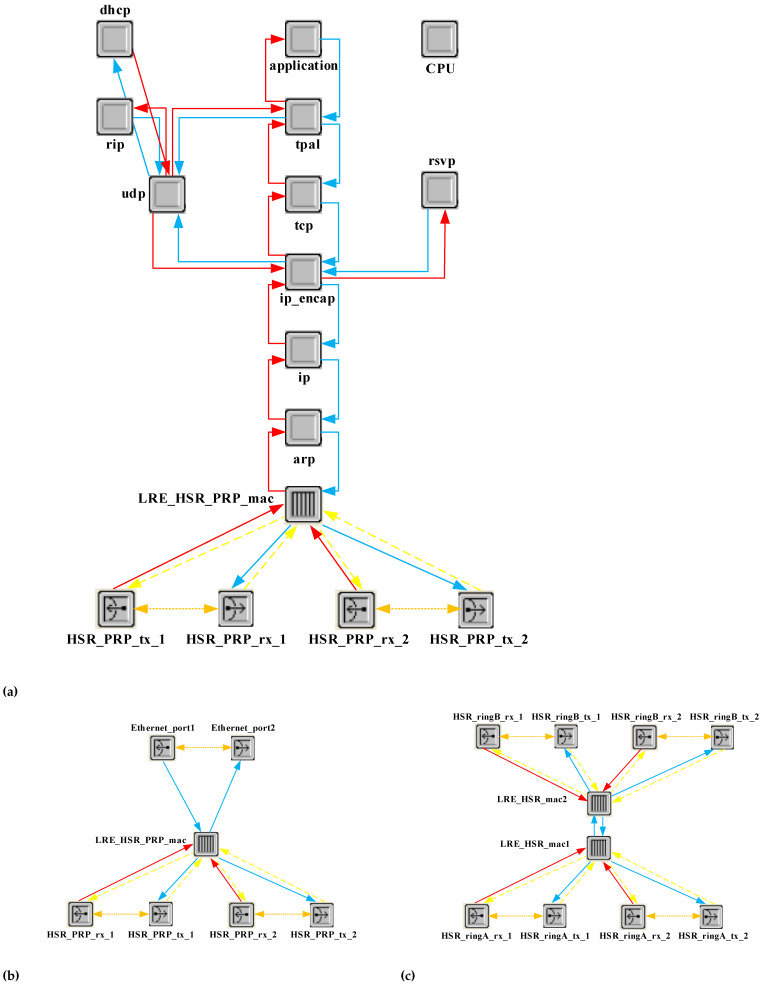
IEC 62439-3 implemented nodes in Modeler. (**a**) DANH and DANP nodes. (**b**) RedBox node in Modeler. (**c**) QuadBox node in Modeler.

To implement DANH and DANP, we deployed ethernet_wkstn_adv, an editable Ethernet workstation in the OPNET Modeler library, as shown in Figure 8a. We designed LRE in the MAC layer of the ethernet_wkstn_adv node, where the hash table registers received frames. The hash algorithm enhances the search time of finding repetitive frames by checking their sequence number. In addition, LRE duplicates received packets from the upper layer and assigns the sequence number to them.

Figure 8b,c illustrate the implementation of RedBox and QuadBox nodes in the OPNET Modeler. MAC tables are installed on both devices, referred to as ProxyNodeTables in their LRE. This MAC table prevents forwarding messages to non-destination PRP LAN or HSR rings. According to the IEC 62439-3, each DANP and DANH in a specified period named LifeCheckInterval multicast PRP_Supervision frame or HSR_Supervision frame including their MAC address, respectively. ProxyNodeTables in RedBoxes and QuadBoxes continue updating the HSR_Supervision frame data by eliminating nodes that do not send supervision frames after a period called NodeForgetTime. This performance by decreasing excessive traffic facilitates extending the size of the network.

### 4.2. Redundant Network Theoretical and Simulation Analysis

Given the ETE delay time according to (1) introduced in Section 2, the following setting is deployed during all simulations. To determine the time limitation of communication between IEDs inside substations, IEC 61850-90-4 limited the $T_{proc}$ to 1.2 ms for each node participating in the substation communication network. Assuming that each DANH hires cut through technology, we assign zero to $T_{sw}$. $T_{q}$ will also be set to zero due to the implementation of the synchronization protocol IEEE 1588 (PTP). According to Table 1, the most strict time was allocated to classes P1 and P7 for GOOSE and SV messages, respectively. Therefore, we consider the maximum allowance of ETE delay for each case study based on a 3 ms latency constraint. The nodes’ communication link is Gigabit Ethernet, with a propagation speed of $2\times 10^{8}$ m/s. The traffic type is SV configured as follows.

(6) $$inter\;arrival\;time_{SV}=\frac{1}{4800}=208.3\;\mu s,$$

(7) $$Traffic_{SV}=\frac{Size\;of\;SV\;(bits)}{inter\;arrival\;time_{SV}}=\frac{180\times 8}{208.3}=6,923,076\;\left(\mathrm{bit}/s\right).$$

We examine the accuracy of implemented IEC 62439-3 nodes by comparing the result of simulation and theory in case study 1. Furthermore, the other scenarios will investigate the different combination performances respecting IEC 61850 messages constraint.

#### 4.2.1. Case Study 1: HSR Ring

**Figure 9:**
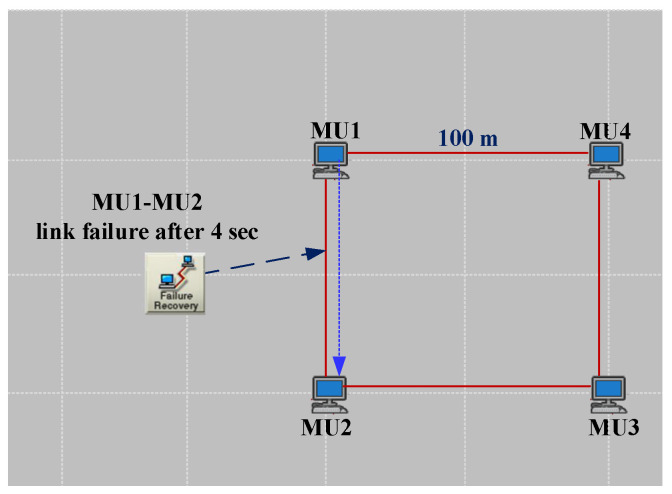
Simple HSR ring (case study 1).

As illustrated in Figure 9, there is an HSR ring, including four DANHs, in this scenario. The traffic is set to SV according to (7). The link is 1 Gb/s Ethernet. Therefore, the $T_{net}$ is calculated as follows in the normal situation operation of the network.

(8) $$T_{net}=T_{prop}+T_{Tran}=\frac{100}{2\times 10^{8}}+\frac{180\times 8}{1\times 10^{9}}=1.94\;\mu s.$$

**Figure 10:**
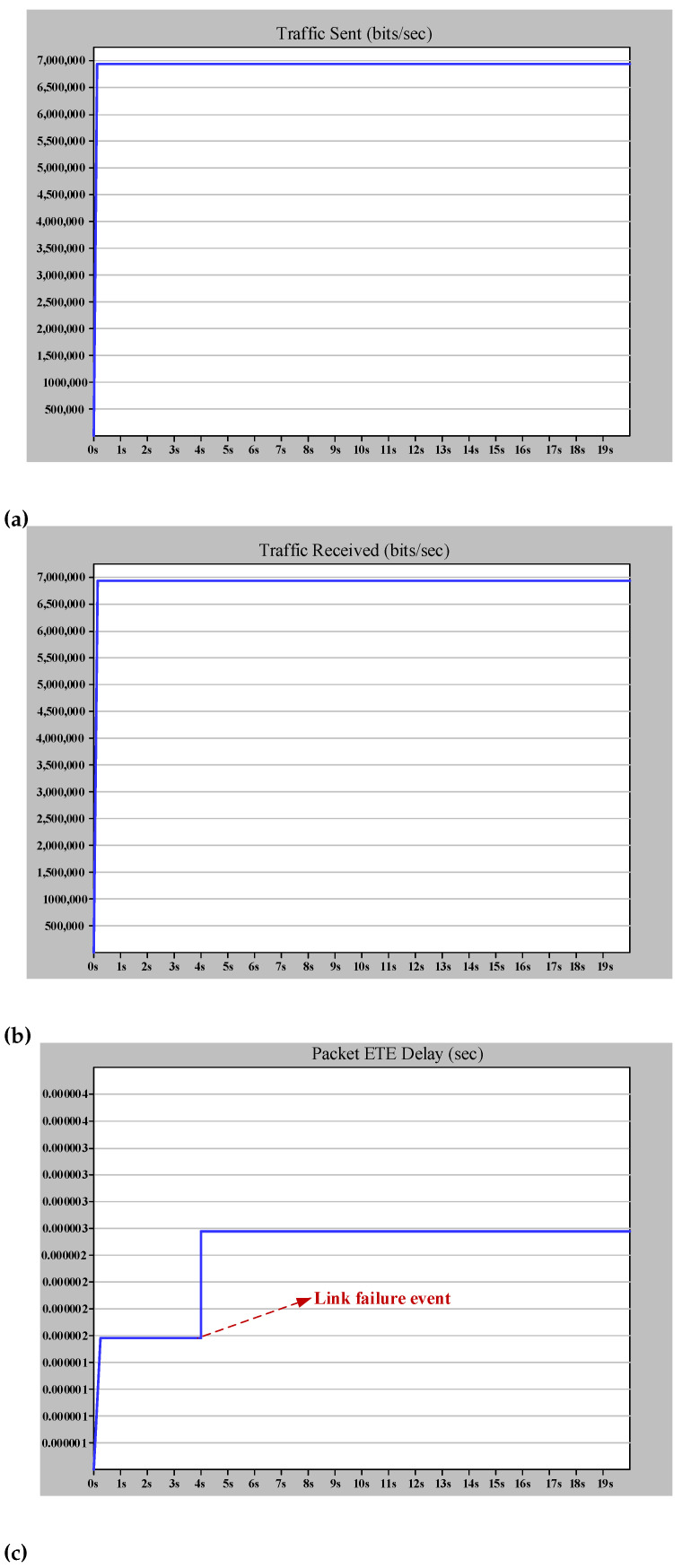
Simple HSR ring simulation results (case study 1). (**a**) The (bits/s) multicast traffic sent by MU1. (**b**) The (bits/s) multicast traffic received by MU2. (**c**) The ETE delay for communication between MU1 and MU2.

The amount of ETE delay represented from theory in (8) is proved by simulation, as shown in Figure 10c. Furthermore, Figure 10a,b demonstrate that the zero recovery arrangement of HSR will prevent any traffic loss due to the link failure. This failure applies to the worst case of link failure that results in the maximum distance between destination and source. From the theoretical point of view, this failure will triple $T_{prop}$ and increase the $T_{net}$ to 3.94 μs. The simulation result in Figure 10 confirms the theoretical analysis and shows the effectiveness of DANH implementation.

According to the simple HSR ring theory results, we determine the maximum number of DANH in the HSR ring. To compute the maximum allowable latency by (9), we consider IEC 61850 standard latency constraints as follows.

(9) $$T_{D,max}=3-2\times 1.2=0.6\;\mathrm{ms}.$$

As a result, (10) describes the maximum number of DANH in an HSR ring.

(10) $$N_{max}-1\leq \frac{T_{D,max}}{T_{prop}\left(N_{max}-1\right)+T_{Tran}},$$

where $N_{max}$ is the maximum number of DANH in the HSR ring. Given the case study 1 results and the worst case of link failure, the $N_{max}$ is equal to 35.

**Figure 11:**
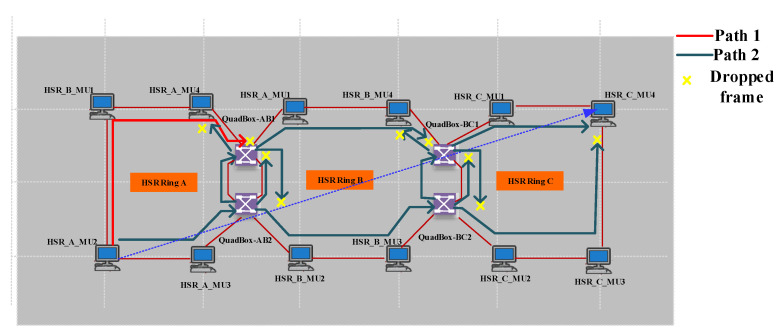
Serial arrangement of HSR rings with QuadBox (Case study1).

**Figure 12:**
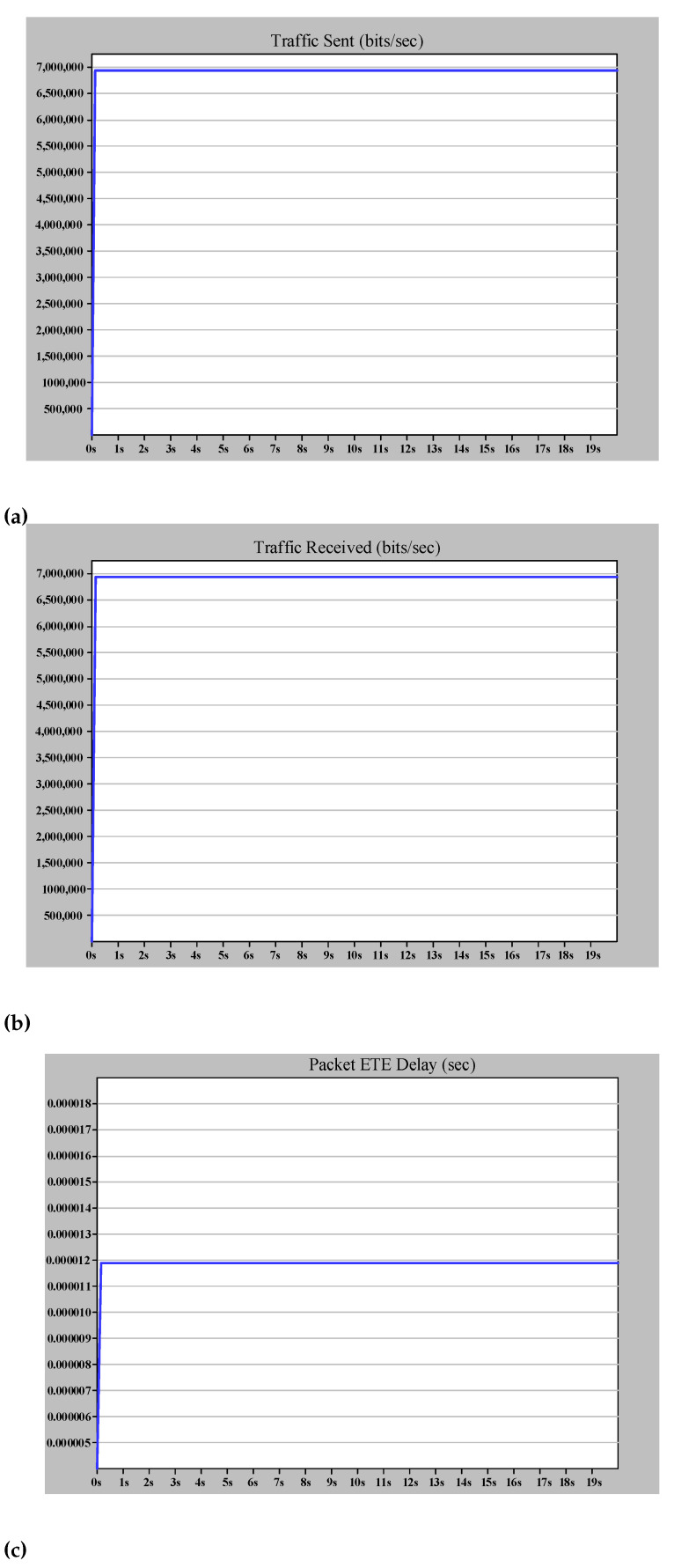
Serial arrangement of HSR rings ETE delay simulation results. (**a**) The (bits/s) multicast traffic sent by HSR_A_MU2. (**b**) The (bits/s) multicast traffic sent by HSR_C_MU4. (**c**) The ETE delay for communication between HSR_A_MU2 and HSR_C_MU4.

**Figure 13:**
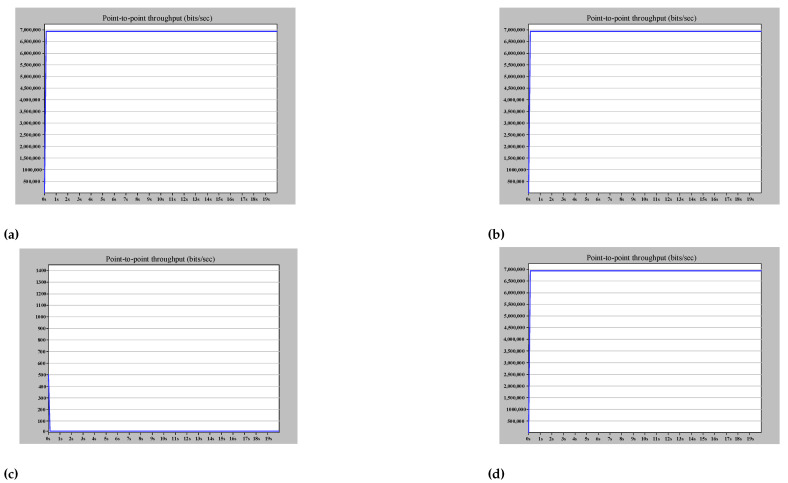
Attached HSR ring QuadBox links throughput simulation results. (**a**) Throughput of QuadBox_AB2–>QuadBox_AB1 in Ring A. (**b**) Throughput of QuadBox_AB1–>QuadBox_AB2 in Ring B. (**c**) Throughput of QuadBox_AB1–>QuadBox_AB2 in Ring A. (**d**) Throughput of QuadBox_AB2–>QuadBox_AB1 in Ring B.

Using QuadBox is also another method for overcoming HSR ring node limitations. Figure 11 is an example of using QuadBoxes to connect HSR rings serially, illustrating in detail the functionality of QuadBox. Whereas Figure 12 shows the good performance of the simulated example when the amount of injected traffic is equal to the received traffic in the destination, Figure 13 shows how the QuadBox blocks the injection of the duplicate frame to the neighbor ring to prevent extra traffic flow. According to Figure 11, since path2 is shorter than path1, its frames reach QuadBox-AB2 earlier and conduct to QuadBoxe-AB1 and Ring B. The QuadBox-AB1 forwards the received frames from its mated QuadBox to Ring B and the neighbor DANH node in Ring A, i.e., HSR_A_MU4. However, the frames drop in HSR_A_MU4 in Ring A since it is a repetitive frame. The QuadBoxe-AB1 drops the frames received from path 1 direction since the duplicate frame was already received from path 2. Therefore, the traffic throughput in the direction from QuadBox-AB1 to QuadBox-AB2 in Ring A is zero, as shown in Figure 13c. Additionally, both QuadBoxes do not forward frames from Ring B to Ring A from their interlink since they have already injected those frames from Ring A to Ring B. This is because QuadBox exhibits the same behavior as a DANH when dropping repetitive frames.

Several configurations of HSR with application of QuadBox can be found in [27,31]. However, although QuadBox improves the system scalability, it will increase the implementation and maintenance cost of the network. Another drawback of attaching HRS rings with QuadBox is that it is prohibited by IEC 62439-3 for use in combination with PRP since this can cause extra frame circulation and traffic flooding.

#### 4.2.2. Case Study 2: Simple PRP

**Figure 14:**
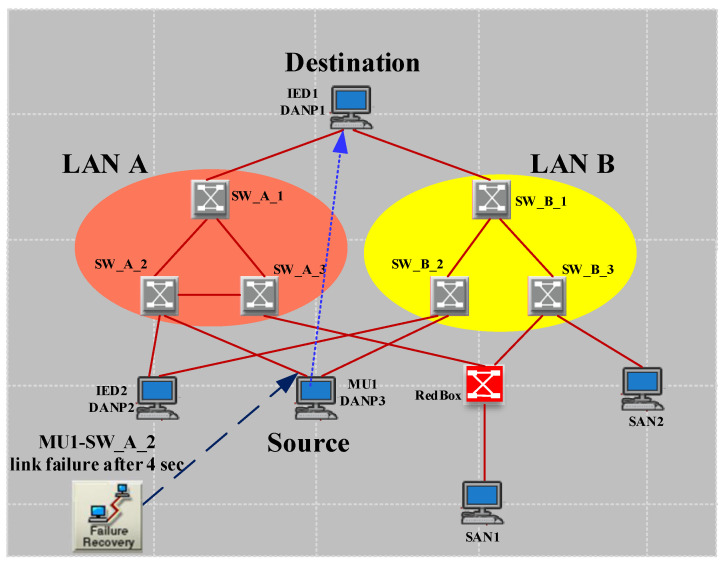
PRP case study 2.

In this scenario, we consider a PRP arrangement according to Figure 14. In PRP, two LANs work in parallel. Therefore, with an increase in the number of switches in each LAN, the PRP supports more IEDs and MUs.

**Figure 15:**
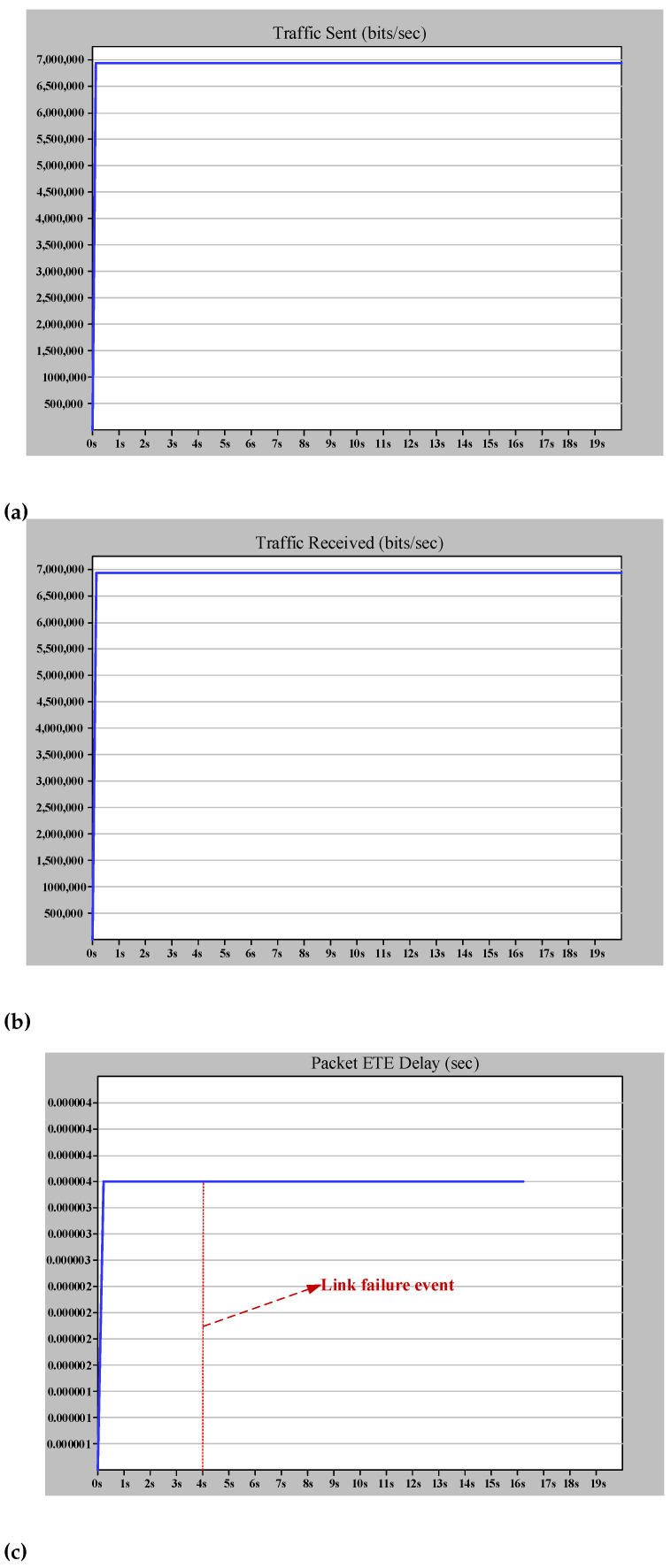
Simple PRP simulation results (case study 2). (**a**) The (bits/s) multicast traffic sent by MU1 (DANP3). (**b**) The (bits/s) multicast traffic received by IED1 (DANP1). (**c**) The ETE delay for communication between MU1 and IED1.

Figure 15 shows the case study 2 simulation results. According to Figure 15a,b, the number of sent and received frames is equal, indicating that the destination only receives the earlier frame and drops the duplicate one. Furthermore, Figure 15c shows that LAN B, which operates independent of LAN A, will transfer frame data without varying ETE delay when there is a link failure between MU1 and SW_A_2. Therefore, PRP provides zero recovery for communication in case of each failure in one of the networks compared to HSR. As shown in case study 1 in HSR, the ring topology provided the recovery path. Hence, the ring path length affected the ETE delay and consequently limited the number of nodes. The only drawback of this excellent performance of PRP is the high expense of providing two distinct LANs.

#### 4.2.3. Case Study 3: PRP and HSR Ring Combination

In this section, we study the configuration of the substation network with the collaboration of HSR and PRP arrangements. In addition to the complexity of extending the HSR ring with QuadBox, the scalability of PRP requires two separate LANs, which means high investment. Therefore, we need a tradeoff between both methods’ pros and cons. It is also notable that IEC 62439-3 standard does not allow using connected HSR rings with QuadBox when HSR combines with PRP.

**Figure 16:**
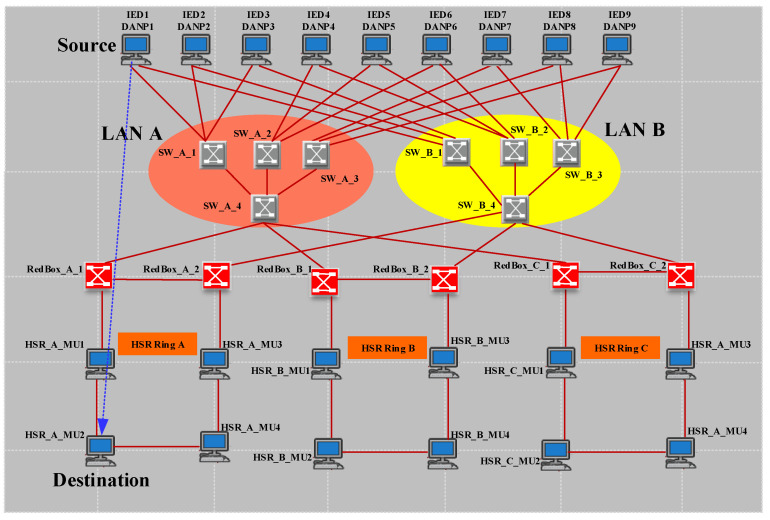
PRP and HSR combination case study 3.

Figure 16 represents an example of these two protocols’ collaboration where PRP is used to provide a redundant station bus network, and HSR connects process bus components. The RedBoxes connect HSR rings to Ethernet switches of the PRP network.

**Figure 17:**
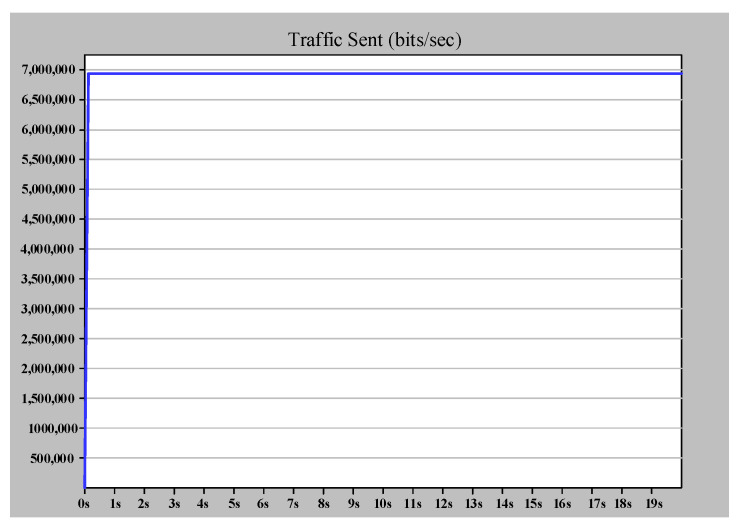

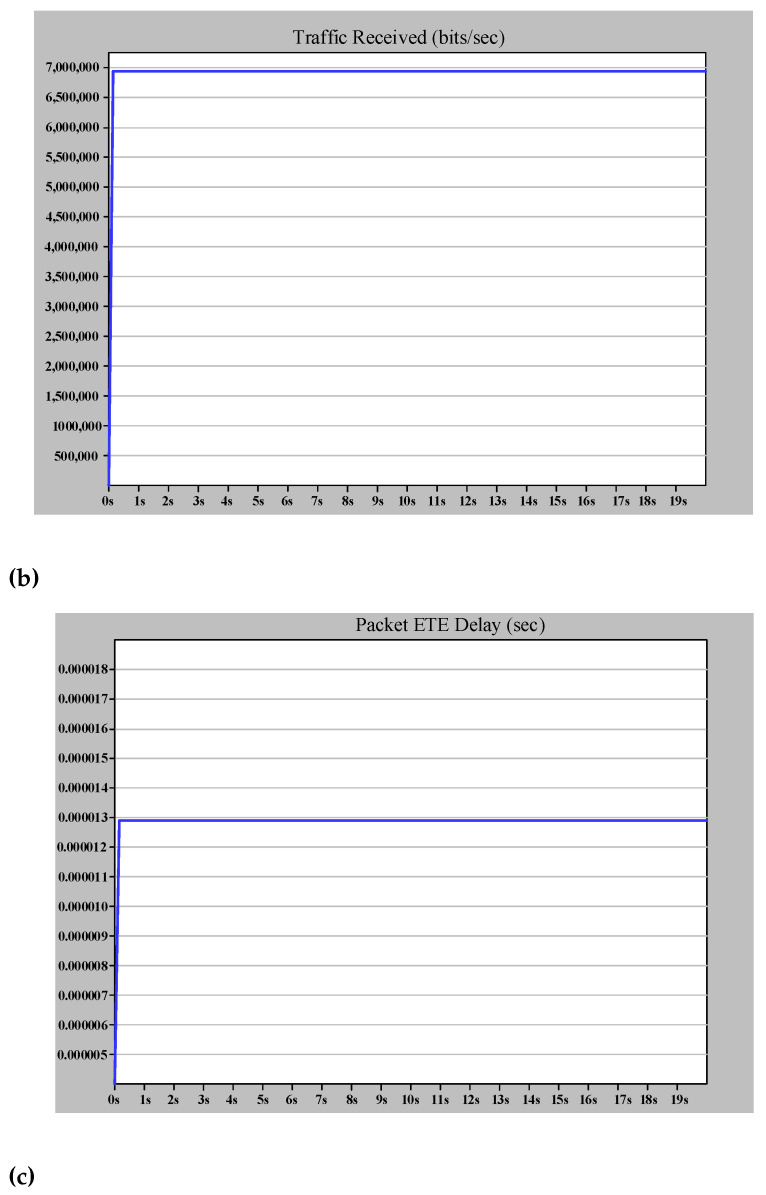
Simple PRP simulation results (case study 1). (**a**) The (bits/s) multicast traffic sent by IED1-DANP1. (**b**) The (bits/s) multicast traffic received by HSR_A_MU2. (**c**) The ETE delay for communication between DANP1 and HSR_A_MU2.

Figure 17 reveals the traffic amount between source and destination and ETE delay.

**Figure 18:**
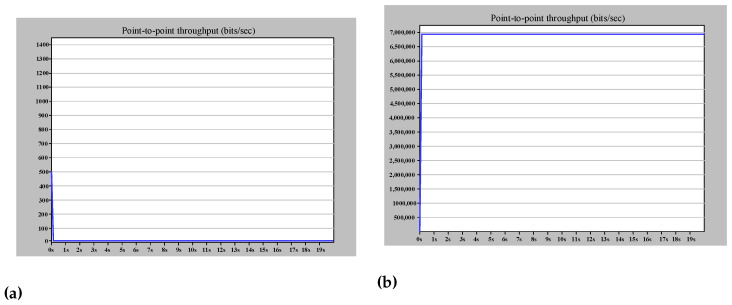

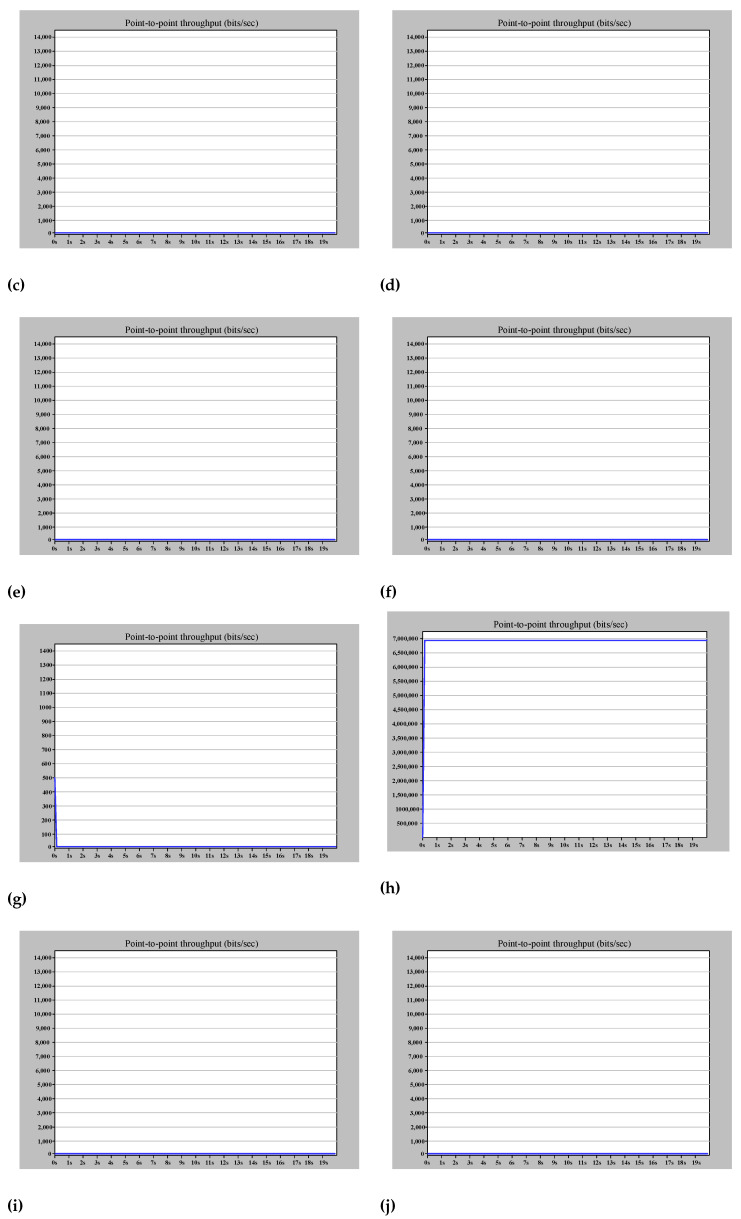

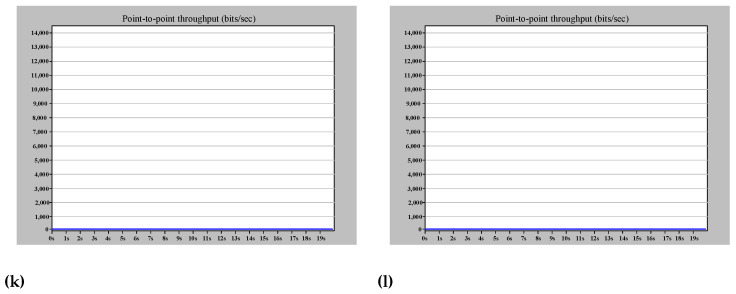
Throughput of links between RedBoxes and switches (case study 3). (**a**) RedBox_A_1 –> SW_A_4 throughput. (**b**) SW_A_4 –> RedBox_A_1 throughput. (**c**) RedBox_B_1 –> SW_B_4 throughput. (**d**) SW_B_4 –> RedBox_B_1 throughput. (**e**) RedBox_C_1 –> SW_C_4 throughput. (**f**) SW_C_4 –> RedBox_C_1 throughput. (**g**) RedBox_A_2 –> SW_A_4 throughput. (**h**) SW_A_4 –> RedBox_A_2 throughput. (**i**) RedBox_B_2 –> SW_B_4 throughput. (**j**) SW_B_4 –> RedBox_B_2 throughput. (**k**) RedBox_C_2 –> SW_C_4 throughput. (**l**) SW_C_4 –> RedBox_C_2 throughput.

Figure 18, which shows the throughput of RedBoxes links, proves the excellent performance of designated RedBox in simulation. By deploying path and network identifiers, frames will not circulate in non-destination HSR rings, thus preventing the excess flow of messages.

## 5. Conclusions

In this paper, we propose the implementation of HSR and PRP protocols as IEC 61850 recommended redundancy arrangements for digital substations. We discussed both the benefits and limitations of each method. The network size was analyzed based on latency of automated substation messages and throughput characteristics. Additionally, this paper examined the IEC 62439-3 constraints and recommendations in each architecture. However, this study requires complementing research by implementing some real examples of substation arrangements which we consider for future work. There are also other methods to extend scalability of the system such as VLAN arrangement which will be deployed in the future work. 

## Figures and Tables

**Table 1:** Substation message specifications based on IEC 61850.

Message Type	Performance Class	Protocol	Max Delay (ms)	Frame Size (byte)	Transmission Period (sample/s)	Throughput (kbps)	Application
	P1		3			1.28	
					1		
GOOSE		Multicast		160	(Stable status)		Protection
	P2		10		32	40.96	
	P3		10–100		(Event status)		
	P4		100				
	P5	IP/TCP	500				Control
	P6		1000				
MMS	P9	IP/TCP/FTP	10,000	256	1	2.048	Management
	P10		500				
	P11	IP	1000				Control
	P12		10,000				
SV	P7	Multicast	3	180	4800	6912	Process Bus
	P8		10		15,360	22,118.4	

## Data Availability

Not applicable.

## Abbreviations

The following abbreviations are used in this manuscript:

CT	current transformers
DANH	doubly attached node HSR
DANP	doubly attached node PRP
DNP3	distributed network protocol 3
ETE delay	end-to-end delay
GOOSE	generic object-oriented substation event
HSR	high-availability redundancy protocol
IED	intelligent electronic devices
IP	Internet protocol
LAN	local area network
LRE	link redundancy entity
MAC	medium access control
MMS	manufacturing message specification
MU	merging unit
PRP	parallel redundancy protocol
RedBox	redundancy Box
RSTP	rapid spanning tree protocol
SAN	single attached nodes
SV	sampled value
TCP	transmission control protocol
VDAN	virtual doubly attached node
VLAN	virtual local area network
VT	voltage transformers
